# Multiple Dynamic Covalent Bond Crosslinked Ionic Liquids-Based Hydrogel with Stretchable, Rapid Self-Healing and Antibacterial Activity Properties

**DOI:** 10.3390/gels12080697

**Published:** 2026-08-04

**Authors:** Ailing Zhang, Xuepeng Wang, Shufen Hou, Guoqing Sui, Kaoxue Li, Shuhua Cao, Panpan Sun

**Affiliations:** 1 College of Chemistry and Chemical Engineering, Weifang University, Weifang 261061, China; 20190020@wfu.edu.cn (A.Z.); houshufen323@163.com (S.H.); gqchem90@163.com (G.S.); likaoxue@wfu.edu.cn (K.L.); shuhua@wfu.edu.cn (S.C.); 2 The Second Team of Hydrogeology and Engineering Geology, Shandong Provincial Bureau of Geology & Mineral Resources (Shandong Provincial Lubei Geo-Engineering Exploration Institute), Dezhou 253072, China; wzxnpn11@163.com; 3 College of Water Sciences, Beijing Normal University, Beijing 100875, China; 4 School of Life Science and Technology, Shandong Second Medical University, Weifang 261053, China

**Keywords:** hydrogels, self-healing, dynamic covalent bonds, antibacterial activity

## Abstract

The development of antibacterial hydrogel with stretchable and self-healing properties is an urgent problem in the field of biomedical engineering. Herein, a series of hydrogels with antibacterial activity was successfully fabricated using polyvinyl alcohol (PVA), borax, 4-formylphenyl-*β*-D-allopyranoside (HLC), 3,3′-dithiobis (propionohydrazide) (DPH) and ionic liquid, 1-aminopropyl-3-methylimidazolium bromide (C_3_MimNBr). The hydrogels were formed via in situ crosslinking through multiple dynamic covalent bonds, primarily including borate ester bonds, imine bonds and acylhydrazone bonds. A Field Emission Scanning Electron Microscope (FE-SEM) revealed that the formed hydrogels possessed a typical three-dimensional network structure. Notably, the interpenetrating network structure endowed the hydrogels with excellent stretchability and self-healing capability, as demonstrated by their ability to be molded into various shapes and stretched up to five times their original length. Furthermore, the mechanical properties of the hydrogel were affected by the amount of the ionic liquid added. Antibacterial evaluation using the colony counting method showed that the hydrogels exhibited outstanding antibacterial activity against *Staphylococcus aureus* (*S. aureus*) and *Escherichia coli* (*E. coli*). In summary, the multifunctional hydrogels, with favorable stretchability and antibacterial activity, represent promising alternative materials for biomedical engineering applications.

## 1. Introduction

Hydrogel is an important class of soft materials with a three-dimensional network structure, which is capable of being widely applied in biomedical fields, such as wound healing, drug delivery, tissue engineering, biosensors, regenerative medicine, antibacterial medical materials [1,2,3,4,5,6], and so forth. Many polymers have been fabricated by a natural polymer, such as polypeptides, hyaluronic acid, cellulose, chitosan, alginate and gelatin, and chemically synthesized compounds, including polyvinyl alcohol, polyacrylic acid and polyethylene glycol [7,8,9]. However, most traditional hydrogels are easily damaged when they undergo external stress, which significantly limits their practicability, particularly in applications such as antibacterial materials.

In recent years, self-healing hydrogels have attracted extensive attention due to their unique ability to repair damage, which can extend the hydrogel lifespan and preserve or restore its original properties. Generally, the self-healing hydrogels can be constructed by intermolecular interactions (mainly including hydrogen bonds, host–guest interactions and ionic bonding) or dynamic covalent bonds. Dynamic covalent bonds, including imine bonds, acylhydrazone bonds, disulfide bonds and borate ester bonds, combine the advantages of tunable weak interactions and the robustness of covalent bonds [10,11,12,13,14]. For instance, Jiang et al. fabricated an ultrafast self-healing hydrogel based on an agarose/PVA via forming double networks [15]. Loos et al. reported a biodegradable and self-healing starch-based hydrogel (starch/polyvinyl alcohol (PVA)/chitosan/borax) by forming dynamic borate ester bonds [16]. Zhou et al. constructed a fast self-healing hyaluronic acid hydrogel with a dual dynamic network, which was a hydrogel that could self-heal rapidly within 30 min at physiological temperature, with a self-healing efficiency of 100%, and that exhibited superior performance in promoting full-thickness skin wound healing [17]. To further functionalize these self-healing hydrogels, the self-healing hydrogels with antibacterial activity have been developed. Currently, antimicrobial activity in hydrogels is achieved mainly through three approaches: (i) the incorporation of inorganic nanoparticles; (ii) the co-assembly of external antibiotics or antimicrobial agents; (iii) the intrinsic antibacterial properties. One of these antibacterial agents, the inorganic nanoparticles, e.g., silver-based, gold-based, metal oxide nanomaterials, such as zinc oxide, iron oxide, etc., usually inflict harm on non-target cells and tissues, and are prone to deposition within various organs. Xiao et al. found that metallic nanomaterials can induce antibiotic resistance, with their physicochemical properties, exposure scenarios and bacterial responses as the major influencing factors [18]. In addition, in antimicrobial drug combination therapy, antibiotics, e.g., tobramycin, kanamycin, amoxicillin, gentamicin etc., can easily accelerate the development of bacterial resistance if they are used improperly. The third category of hydrogel building blocks with intrinsic antibacterial activity mainly includes polypeptides and cationic polymers (such as quaternary ammonium salts and quaternary phosphonium salts). These components exhibit excellent antibacterial efficacy and are unlikely to induce increased bacterial drug resistance. Compared with antibacterial polypeptides, quaternary ammonium and quaternary phosphonium-based cationic polymers boast simple synthesis steps and mature purification and scale-up technologies, which has led them to become a mainstream antibacterial component for fabricating antibacterial hydrogels in recent years [19].

**Scheme 1:**
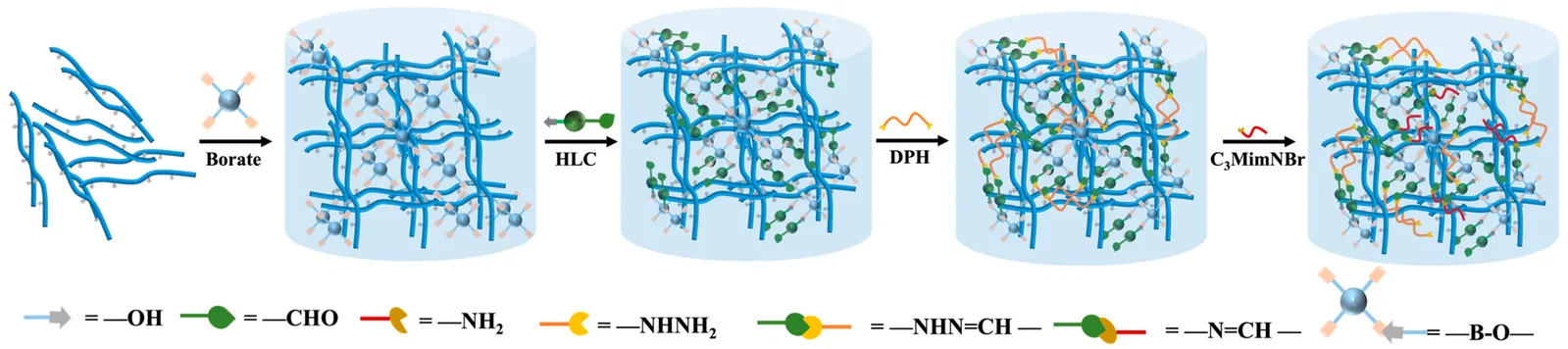
Schematic diagram of the formation mechanism of hydrogel through multiple dynamic covalent bonds.

Ionic liquid derivatives (ILDs) are a promising class of antimicrobial agents in the fields of disinfectants and cleaning agents in food and pharmaceutical industries and hospitals [20]. Various cationic of ILDs with different antibacterial activities, such as ammonium, imidazolium, pyridinium, and phosphonium, have been reported [21,22,23,24,25,26,27,28]. They differ from conventional antibiotics in that their bactericidal activity is primarily based on physical membrane disruption [29,30]. Xu et al. designed a series of flexible fluorescent diketopyrrolopyrrole-based imidazolium ILDs, and systematically studied the structure–antibacterial activity relationships of the ILDs against *Escherichia coli* (*E. coli*) and *Pseudomonas aeruginosa* (PAO1). They found that with the increase in molecular sizes, the corresponding ILDs intercalated into the bacterial membrane, leading to the destabilization of the lipid bilayer and further contributing to the antimicrobial activities [31]. In addition, Dastidar et al. designed a series of primary ammonium sulfonate salts (A*_n_* = CH_3_-(CH_2_)*_n_*-NH_2_; *n* = 2–11, 13–15, 17) and verified that the salt A_14_N2S had the best ability to kill the Gram-positive bacterium *Staphylococcus aureus* [32]. With the advancement of related research, ILDs have gradually become one of the important materials for the construction of antibacterial hydrogels in recent years. Zheng et al. introduced pyrrolidinium ionic liquid (C*_n_*MPBr) into polyvinyl alcohol–tetrahydroxyborate anion (B(OH)_4_^−^) system via electrostatic interaction and successfully constructed a hydrogel with self-healing properties and antibacterial activity [33]. Li et al. fabricated a novel non-releasing antibacterial hydrogel by methyl methacrylate, 1-vinyl-3-butylimidazolium, polyethylene glycol dimethacrylate, acrylamide and *N*,*N*-methylene-bis-acrylamide, and confirmed the antibacterial property of hydrogel was attributed to the ionic liquid of 1-vinyl-3-butylimidazolium [34]. Therefore, the introduction of ionic liquids into the hydrogel system could endow the hydrogel with antibacterial activity. However, the covalent incorporation of ionic liquids into the system typically requires complicated synthetic procedures, including the use of catalysts. Introduction via non-covalent bonds suffers from the drawback of facile release or leakage. In contrast, the introduction of dynamic covalent bonds as crosslinking points offers the advantages of low cost and facile preparation. In this work, the hydrogel was successfully fabricated by polyvinyl alcohol (PVA), borax, 4-formylphenyl-*β*-D-allopyranoside (HLC), 3,3′-dithiobis(propionohydrazide) (DPH) and ionic liquid, 1-aminopropyl-3-methylimidazolium bromide (C_3_MimNBr) (Scheme 1). The microstructural, rheological, self-healing, stretching and antibacterial properties of the synthesized hydrogel were analyzed. The hydrogels exhibited rapid self-healing ability, excellent stretchability, and good antibacterial activity. Hence, the multifunctional hydrogels had outstanding antibacterial activity, making them a potential candidate for broad-spectrum antibacterial material applications.

## 2. Results and Discussion

### 2.1. Preparation and Characterization of Hydrogels

**Figure 1:**
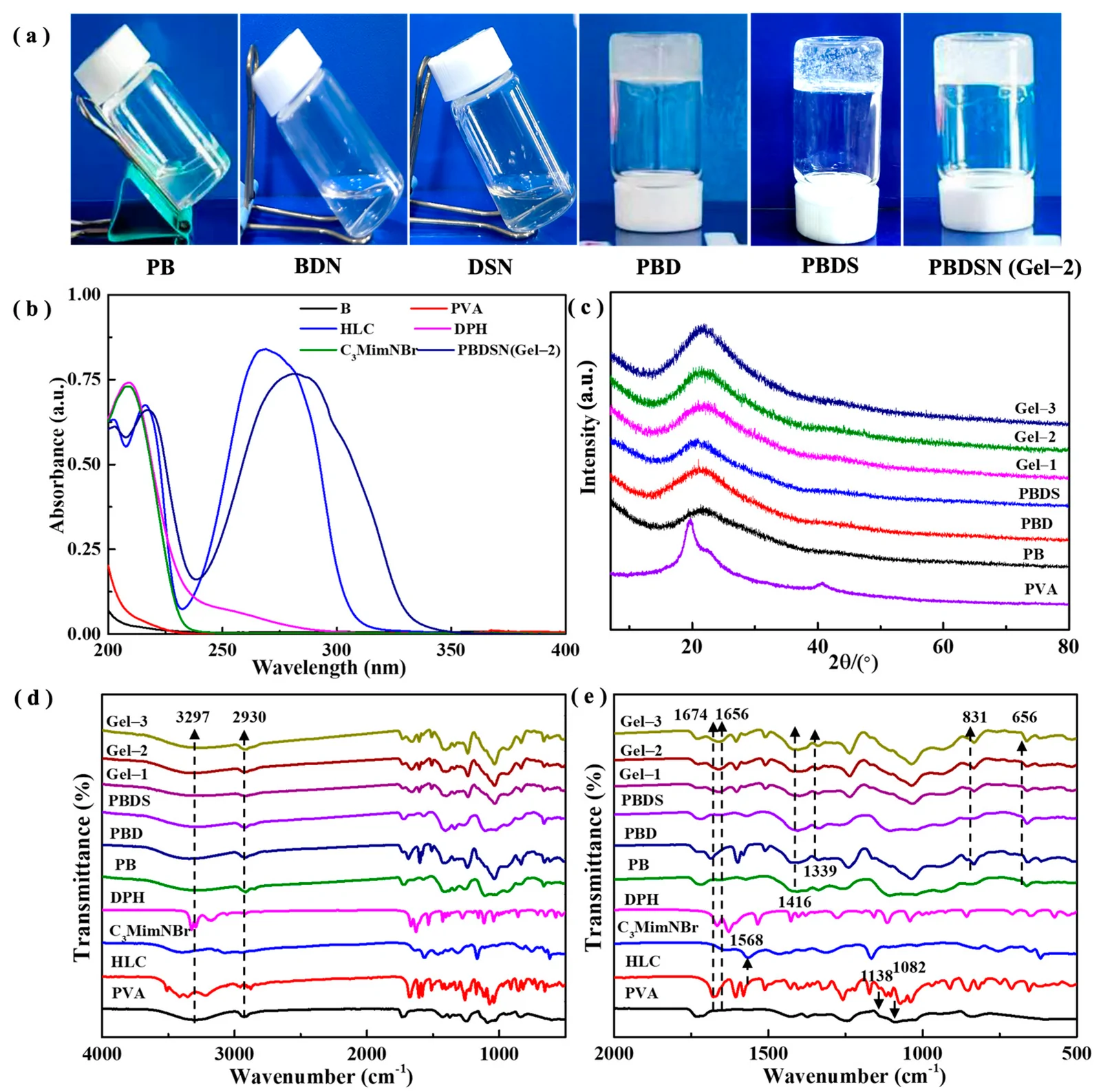
(**a**) The digital photos of samples; (**b**) UV-Vis spectra of components; (**c**) XRD patterns of hydrogels and components; (**d**,**e**) FTIR spectra of hydrogels and components.

The composite hydrogels were fabricated using polyvinyl alcohol (PVA), borax, 4-formylphenyl beta-D-allopyranoside (HLC), 3,3′-dithiobis (propionohydrazide) (DPH) and ionic liquid, 1-aminopropyl-3-methylimidazolium bromide (C_3_MimNBr), through multiple dynamic covalent bonds. The successful formation of the hydrogels was confirmed by the tube inversion non-flow phenomenon (Figure 1a). The UV-Vis spectra of the native PVA, borax (B), HLC, DPH, C_3_MimNBr and the resulting hydrogels (PBDSN) were recorded. As can be seen from Figure 1b, PVA and B exhibit nearly no significant UV-Vis absorption across the scanned range. The absorption bands of C_3_MimNBr and DPH in the 200–210 nm were attributed to the π→π* transition of imidazole ring and C=O group, respectively. For HLC, the adsorption band at 210 nm and 275 nm corresponds to the π→π* transition of C=O group and the benzene ring [35]. Notably, compared with the absorption curves of HLC, those of PBDSN show a distinct bathochromic shift, which may be attributed to the enhanced conjugation following the formation of Schiff base and acylhydrazone bonds during the crosslinking reaction between amino (NH_2_) groups of C_3_MimNBr and acylhydrazine (NH_2_-NH-C=O) groups with aldehyde (CH=O) groups of HLC. Meanwhile, a new absorption peak appeared at approximately 310 nm, likely arising from the n→π* transition of the C=N bonds in the formed PBDSN (Gel-2) [35].

The formation of dynamic covalent bonds, such as Schiff bases, acylhydrazone bonds and borate ester bonds, can be further verified by the FTIR spectra. As depicted in Figure 1e, the stretching vibration peaks corresponding to C=O groups on HLC at 1674 cm^−1^ and NH_2_ groups on C_3_MimNBr at 1568 cm^−1^ disappeared, while a new peak of C=N groups appears at 1656 cm^−1^ in the spectra of PBDS and PBDSN (Gel-1, Gel-2, Gel-3), which can be attributed to the formation of the Schiff bases and dynamic acylhydrazone bonds. Additionally, it was observed the B-O-C asymmetric stretching vibration peaks at 1416 cm^−1^ and 1339 cm^−1^ and B-O-B stretching vibration peaks at 656 cm^−1^ in PB, PBD, PBDS and PBDSN (Gel-1, Gel-2, Gel-3) hydrogels, which confirmed the formation of the borate ester bond [36]. As shown in Figure 1d, the characteristic peaks of PVA near 3297 cm^−1^ and 2930 cm^−1^ can be attributed to the stretching vibration of C-H and O-H groups, which confirmed the existence of PVA in the formation of the hydrogels. Meanwhile, upon the addition of DPH, C_3_MimNBr and HLC, the broad peak at 3297 cm^−1^ shifts to 3325 cm^−1^, which is attributed to intermolecular hydrogen bonding among PVA, DPH, C_3_MimNBr and HLC.

To explore the crystal structure changes before and after gelation, XRD measurements were employed. As can be seen from Figure 1c, the XRD pattern of PVA displays peaks at 2θ = 19.6°, 22.8° and 40.7°, which correspond to the (101), (200), and (102) planes of the PVA crystallites [37]. For lyophilized hydrogels, the sharp diffraction peak at 19.6° turned into a broad peak, and the diffraction peaks at 22.8° and 40.7° disappeared, which probably attribute to the hydrogen bonding, borate ester bonds, amide bonds and acylhydrazone bonds between PVA, borax, DPH, C_3_MimNBr and HLC. The multiple interactions restrict the mobility of molecular chain segments, thereby leading to the amorphous process [37].

### 2.2. Structural Characterization of Hydrogels

**Figure 2:**
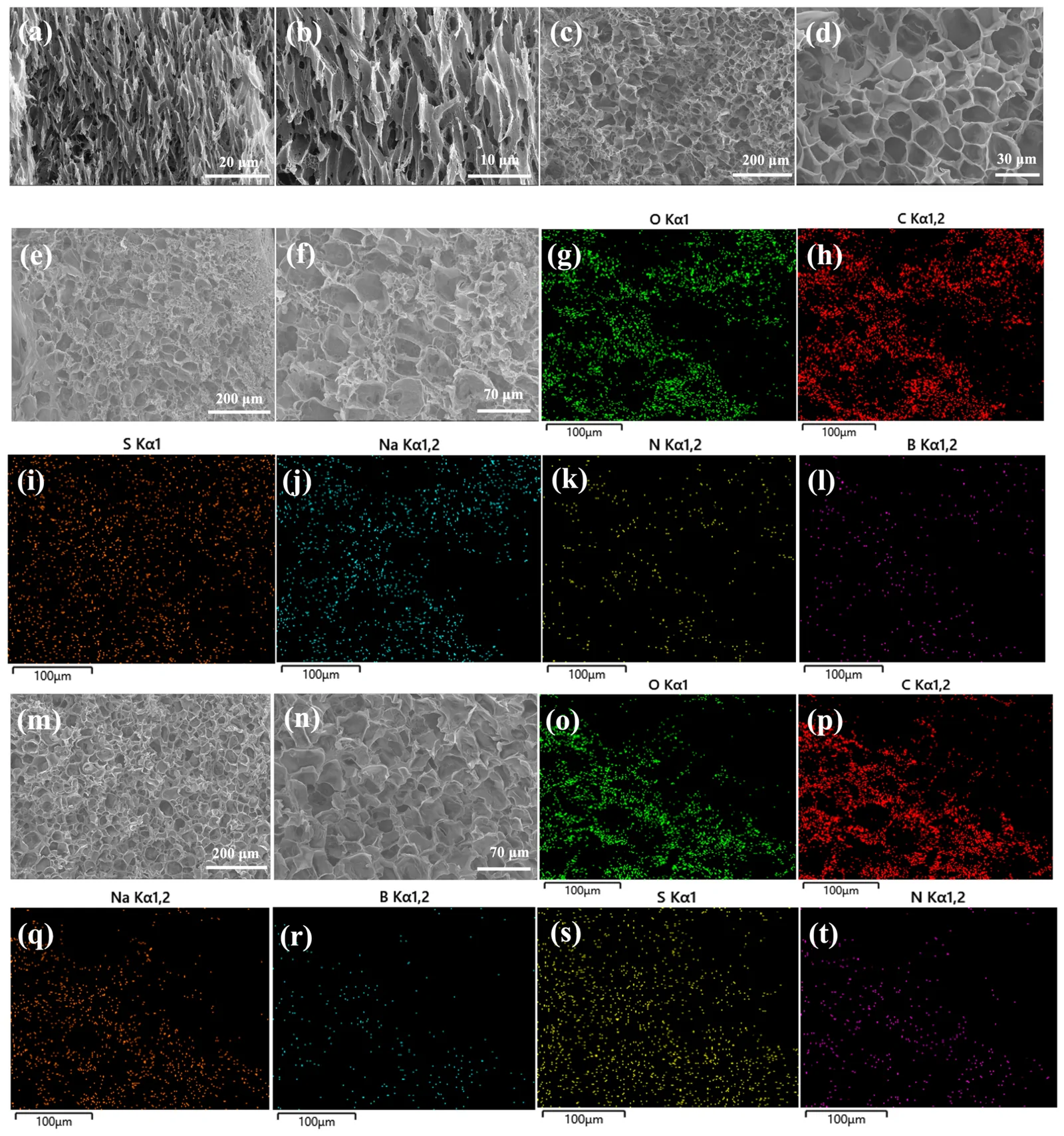
FE-SEM images and mapping images of freeze-dried hydrogels: (**a**,**b**) PB, (**c**,**d**) PBD, (**e**–**l**) PBDS and (**m**–**t**) PBDSN (Gel-2).

The cross-sectional morphologies of the freeze-dried hydrogels were characterized by FE-SEM. As shown in Figure 2a,b, the PB hydrogel exhibited loose sheet-like morphology with an irregular porous structure, which is primarily attributed to the weak hydrogel network resulting from the low concentration of PVA with borax. Compared with PB hydrogel, the PBD hydrogel shows a well-defined three-dimensional network structure. After the addition of DPH and C_3_MimNBr, the PBDS and PBDSN (Gel-2) maintain the interconnected network structure and the mapping images show a uniform distribution of each element in the hydrogels. Obviously, the pore size of PBDS is notably smaller than that of PBD, a change that is ascribed to the formation of dynamic acylhydrazone bonds between the hydrazine of DPH and the aldehyde groups of HLC, which introduced additional crosslinking sites and thereby reinforced the gel network [37]. In contrast to PBDS, the pore sizes of PBDSN (Gel-2) hydrogel displayed an increased pore size. This increase is probably attributed to the introduction of ionic liquid C_3_MimNBr, which participated in the formation of movable dynamic imine bonds (anchored at only one end), while simultaneously reducing one immovable dynamic acylhydrazone bond (tethered at both ends). Consequently, the gel network became looser, leading to an enlarged pore size.

### 2.3. Rheological Properties of Hydrogels

**Figure 3:**
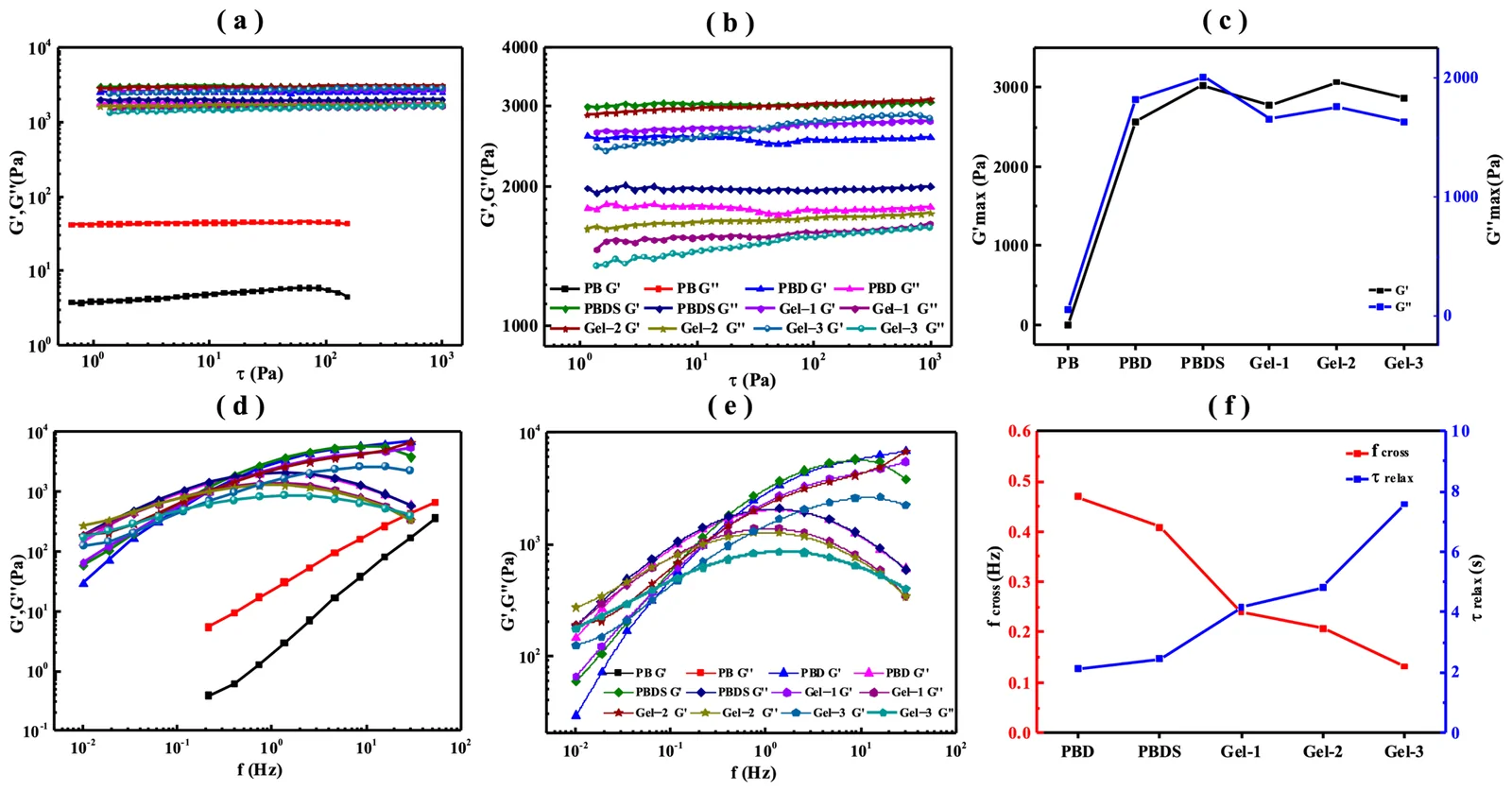
Dynamic viscoelasticity performance of hydrogels: (**a**,**b**) stain sweep tests (1–1000 Pa), (**c**) G′_max_ and G″_max_, (**d**,**e**) frequency sweep tests (0.01–100 Hz) and (**f**) f_cross_ and τ_relax_ for hydrogels.

To further characterize the mechanical properties of the resulting hydrogels, rheological measurements were systematically performed. In stress sweep measurements, as shown in Figure 3a,b, the PB sample exhibited a storage modulus (G′) consistently lower than its loss modulus (G″) throughout the entire strain sweep range, exhibiting typical fluid-like rheological behavior. In contrast, the PBD, PBDS, and PBDSN hydrogels (Gel-1, Gel-2 and Gel-3, respectively) showed G′ > G″ throughout the strain sweep, with the moduli remaining independent of the applied strain, indicative of a solid-like viscoelastic response. The plateau value of the storage modulus obtained at high angular frequencies (G′_max_) represents the intrinsic stiffness of the gel network structure. Compared with PB, the G′_max_ values of PBD and PBDS were markedly increased (Figure 3c), suggesting that the incorporation of HLC and DPH enhanced intermolecular chain entanglements and promoted the formation of a more robust network structure. However, upon the addition of the ionic liquid C_3_MimNBr content from 0.02 g (Gel-1) to 0.05 g (Gel-3), the G′_max_ value decreased, suggesting that C_3_MimNBr partially disrupted the PBDS network structure [36]. At moderate loadings (0.03 g Gel-2), the storage modulus (G′) and loss modulus (G″) have relativity higher values, which may be attributed to the C_3_MimNBr enhanced physical chain entanglement and may have contributed to a more optimized cross-linking topology. As can be seen from Figure 3a, the G′ values of all the samples were relatively stable within τ = 1 Pa, so τ = 1 Pa was applied to ensure that each test was conducted within the linear viscoelastic region. The frequency dependence of G′ and G″ for all samples measuring over the range of 0.01–100 Hz is presented in Figure 3d,e. Apparently, all samples displayed a loss modulus dominant response (G″ > G′) at the low frequency region, indicative of viscous liquids property. Upon increasing the frequency, the G′ of PBD, PBDS, and PBDSN hydrogels gradually approached G″ even to greater than G″, displaying frequency-dependent moduli, which is consistent with the typical rheological signature of the gels with reversible crosslinks and further confirmed the formation of the dynamic covalent bonds [37]. Hydrogels with lower f_cross_ values and higher τ_relax_ values tend to show more elastic nature and better shape stability, reflecting a more stable network structure [12]. As depicted in Figure 3f, the longer τ_relax_ observed for PBD and PBDS relative to PB points to the network reinforcing the role of dynamic covalent bonds, which effectively enhanced the internal structural of the hydrogel. However, the incorporation of the ionic liquid partially reduced the τ_relax_ of the hydrogels, demonstrating a disruptive effect of C_3_MimNBr on the PBDS hydrogel network.

### 2.4. Stretchable, Self-Healing and Shapeable Properties of PBDSN (Gel-2) Hydrogels

**Figure 4:**
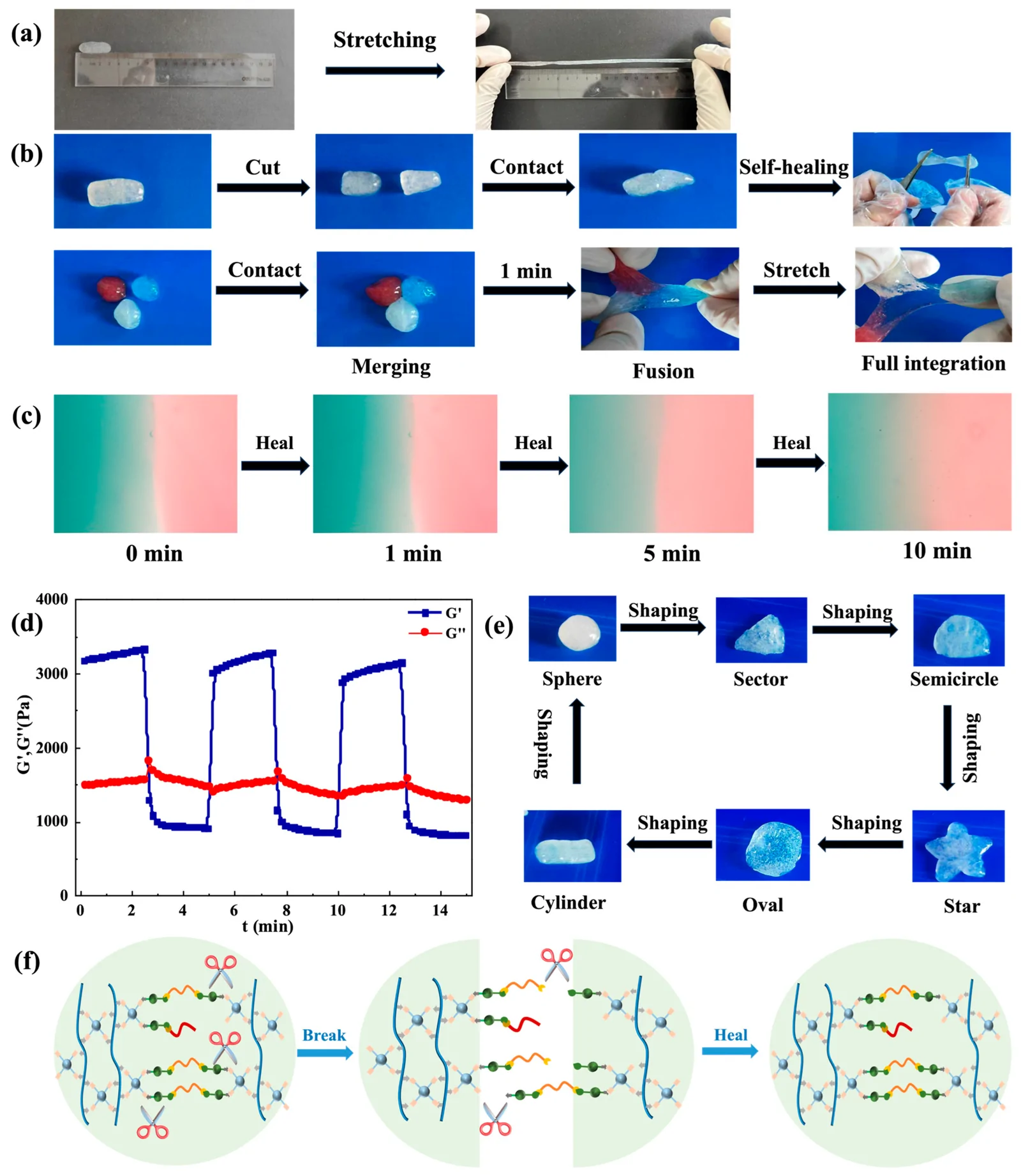
(**a**) Macroscopic stretchable, (**b**) self-healing and (**c**) microscopic self-healing image within 5 min of the PBDSN (Gel-2) hydrogel; (**d**) rheological properties of PBDSN (Gel-2) hydrogel with alternate step strain between 10% and 200% in three cycles; (**e**) macroscopic shapeable of PBDSN (Gel-2) hydrogel; (**f**) the self-healing mechanism of PBDSN (Gel-2) hydrogel.

Hydrogels with appropriate mechanical strength, particularly those combining good stretchability and self-healing ability, are essential to enable rapid recovery after damage and ensure sustained protection of the wound site [38,39]. The stretchable, self-healing and shapeable abilities were investigated and PBDSN (Gel-2) was selected as a representative hydrogel. As shown in Figure 4a, the PBDSN (Gel-2) hydrogel could be stretched to over five times its initial length without fracture, demonstrating excellent stretchability and suggesting its potential application as a functional material. As can be seen from Figure 4b, two stained hydrogel pieces and one unstained piece were gently placed together and rapidly fused into a unified hybrid hydrogel. The integrated hydrogel maintained structural integrity under stretching manipulation. Remarkably, it could be elongated into a membrane, exhibiting the fast self-healing performance of the PBDSN (Gel-2) hydrogel. Meanwhile, the healing status of the two stained hydrogels was observed by optical microscopy. It is obvious that the cracks between two stained hydrogels gradually became shallower within 10 min until they completely disappeared (Figure 4c). To further verify the self-healing ability of the PBDSN (Gel-2) hydrogel, the storage modulus (G′) and loss modulus (G″) were systematically evaluated. As shown in Figure 4d, a dynamic strain cycle test was performed by applying alternating strains of 10% and 200%. Upon application of 200% strain, the G′ value decreased from about 3000 Pa to about 950 Pa and became lower than G″, indicating disruption of the hydrogel network. Notably, when the strain was switched from high strain (200%) back to low strain (10%), the damaged network structure within the hydrogel was rapidly reconstructed and largely restored to its initial mechanical state. Quantitative analysis revealed that the recovery percentages of G′ after the first and second cycles were 94% and 89%, respectively. These results indicate that the PBDSN (Gel-2) hydrogel is capable of maintaining a high structural recovery efficiency even after experiencing large-strain deformation, which fully demonstrates its outstanding self-healing performance.

As shown in Figure 4f, a plausible self-healing mechanism of the hydrogel was proposed. The network of the hydrogel was constructed through multiple reversible interactions, mainly including dynamic Schiff base bonds (formed via the reaction between aldehyde groups of HLC and amino groups of DPH), borate ester bonds (generated from the hydroxyl groups of PVA, HLC and borax), hydrogen bonds (formed between free hydroxyl and amino groups), and electrostatic interactions (formed between borate anions and imidazolium cations) [40]. Upon exposure to external forces, the network structure was disrupted, while the reversible nature of the dynamic imine and borate ester bonds enabled rapid structural recombination, allowing the PBDSN (Gel-2) hydrogel to spontaneously revert to its original state. These results demonstrated the outstanding self-healing capacity of the PBDSN (Gel-2) hydrogel. The shapeable property of the PBDSN (Gel-2) hydrogel was evaluated by means of macroscopic observations, as illustrated in Figure 4e. It was found that the prepared PBDSN (Gel-2) hydrogel could be readily molded and subsequently remolded into well-defined shapes, including sphere, semicircle, sector, star, oval and cylinder, indicative of its excellent shapeable ability, which is a desirable feature for materials requiring flexible processing and shape adaptability.

### 2.5. Antibacterial Performance of Hydrogels

**Figure 5:**
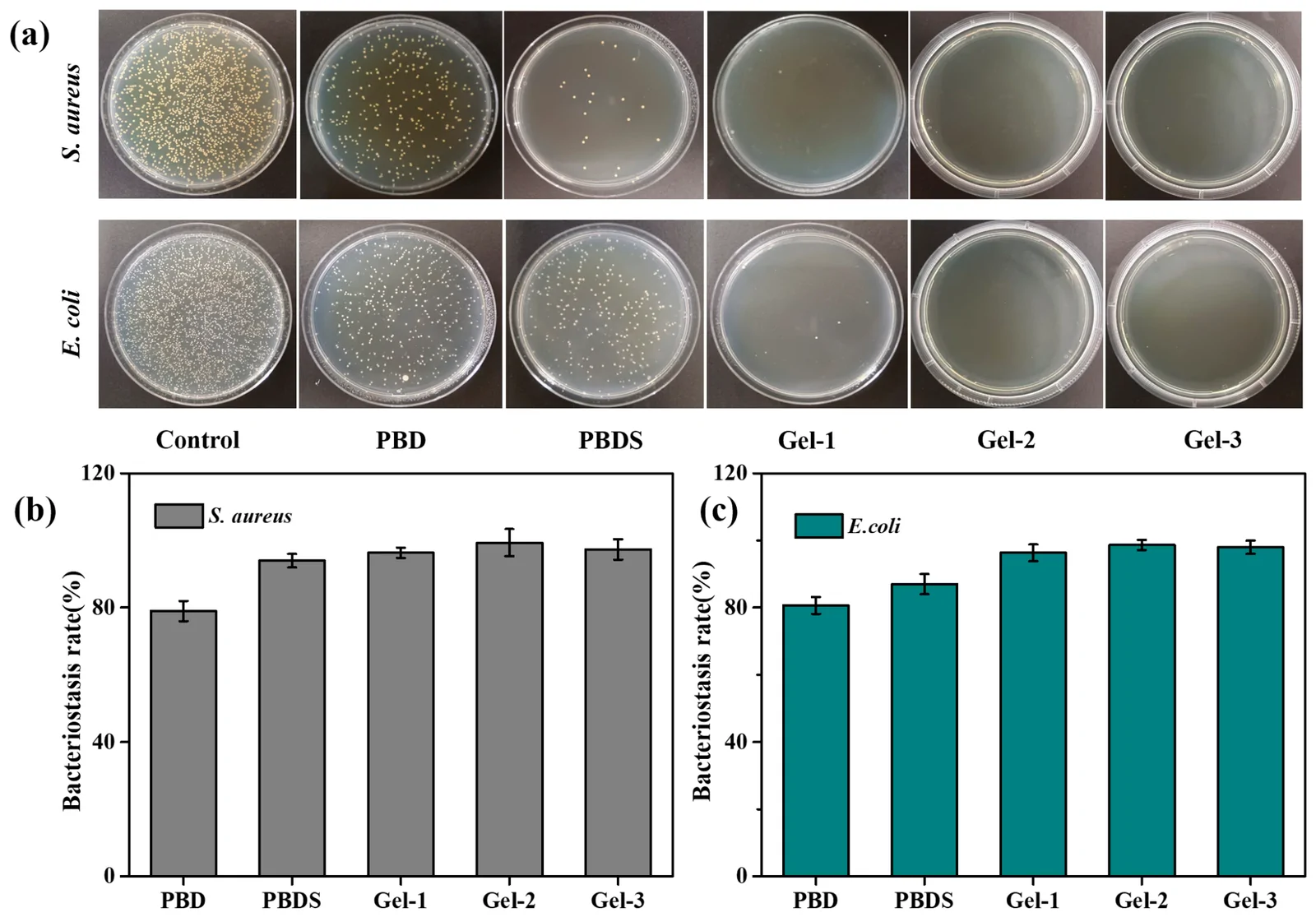
(**a**) Digital photos of the growth of *S. aureus* and *E. coli* cultured in vitro on agar plates under treatments with different hydrogels. Bacteriostasis rates against (**b**) *S. aureus* and (**c**) *E. coli*. Error bars represent standard deviation and reflect the degree of data dispersion.

Bacterial infections can not only hinder the healing process of wounds but also trigger local inflammation. Therefore, the hydrogel with inherent antibacterial activity has become a potential candidate against bacterial infection [41,42]. The antimicrobial performance of the fabricated hydrogels was evaluated using the plate counting method, which was used to evaluate the antibacterial activity of the hydrogels against Gram-positive bacteria *Staphylococcus aureus* (*S. aureus*) and Gram-negative *Escherichia coli* (*E. coli*), respectively. As depicted in Figure 5a, in comparison to the control group, the number of bacterial colonies on plates supplemented with PBDS hydrogel was significantly lower. The PBDSN (Gel-1) hydrogel exhibited complete inhibition (100%) against both *E. coli* and *S. aureus* (Figure 5b,c). It can be seen that the hydrogel has a good antibacterial effect on both bacteria, which is attributed to the inherent antimicrobial properties of both the –NH groups in DPH and the quaternary ammonium moieties of imidazolium-based ionic liquids, with the antibacterial efficacy increasing proportionally with sample concentration. Our hydrogel exhibited significantly higher antibacterial performance, highlighting its strong potential as an effective antibacterial material.

## 3. Conclusions

In summary, a series of PBDSN (Gel-1, Gel-2, Gel-3) hydrogels with good antibacterial activity and mechanical properties was successfully prepared through the one-pot method, and their physicochemical and biological properties were investigated. The formation of dynamic imine and borate ester bonds was confirmed by UV-Vis and FTIR spectroscopy. FE-SEM images showed the three-dimensional porous structure in the hydrogels. The rheological experiments revealed the viscoelastic and thixotropic nature of the hydrogels. In addition, the fractured hydrogel could heal completely within 10 min without any external force, and that could be stretched to over five times its initial length without fracture. The reversible Schiff base bonds formed between the aldehyde groups of HLC and the amino groups of DPH, together with hydrogen-bonding interactions and electrostatic attractions between borate anions and imidazolium cations, collectively endow the PBDSN hydrogels with excellent self-healing performance. Finally, the hydrogels were efficient against both Gram-positive (*S. aureus*) and Gram-negative (*E. coli*) bacteria. Thus, these results demonstrate that the PBDSN hydrogel possesses favorable self-healing capability and antibacterial activity, positioning it as a promising candidate for antibacterial material design.

## 4. Materials and Methods

### 4.1. Materials

Poly(vinyl alcohol) (PVA, alcoholysis: 85.0–90.0 mol%, viscosity: 20.0–30.0 mPa) and 4-formylphenyl-*β*-D-allopyranoside (HLC, purity ≥ 98%) were purchased from Tianjin Xiensi Biochemical Technology Co., Ltd. (Tianjin, China). 1-aminopropyl-3-methylimidazolium bromide (C_3_MimNBr, purity ≥ 98%) was acquired from Titan Technology Exploration Platform (Shanghai, China). Borax (sodium tetraborate decahydrate, purity ≥ 99.5%) was purchased from Sinopharm Group Chemical Reagent Co., Ltd. (Shanghai, China). All chemicals were used as received without further purification. Deionized water was used throughout all the experiments (pH = 6.8).

### 4.2. Synthesis of 3,3′-Dithiobis(Propionohydrazide) (DPH)

DPH was synthesized according to our previous work with slight modifications [43]. In brief, 3.0 g (12.8 mmol) of 3,3′-dithiopropionic acid dimethylester was dissolved in MeOH (50 mL) and stirred slightly for 10 min. Then, 3.150 g of 50 wt% hydrazine hydrate (32 mmol, 2.5 equiv.) was added to the blend dropwise, and the mixture was stirred at room temperature for 3 h. Next, the reaction was refluxed with stirring under N_2_ for 12 h. Upon completion, a white solid precipitated out. The crude product was collected by vacuum filtration and washed with cold MeOH and diethyl ether, three times respectively. The washed solid was then transferred to a vacuum drying oven and dried under reduced pressure at 25 °C for 24 h to afford the purified target product, ^1^H NMR (400.13 MHz, DMSO-*d*_6_) *δ*: 2.33 (t, 2H), 2.84 (t, 2H), 4.14 (s, 2H), 8.87 (s, 1H).

### 4.3. Preparation of the Hydrogel

The hydrogel was prepared by using one-pot method: firstly, PVA solid block (10.0 wt%) was added to deionized water under vigorous stirring at 90 °C until the homogeneous solution formed. Then, the borax, 4-formylphenyl-*β*-D-allopyranoside (HLC), 3,3′-dithiobis(propionohydrazide) (DPH) and ionic liquid, 1-aminopropyl-3-methylimidazolium bromide (C_3_MimNBr), were mixed in PVA aqueous solution with agitating until the solid dissolved thoroughly, and left to cool down to room temperature. After about 2 min, the transparent hydrogel was obtained and named as PBDSN. The PBDSN hydrogels were denoted as Gel-1, Gel-2, and Gel-3 according to the different ionic liquid loadings. (PBDSN: 10.0 wt% PVA, 0.5 wt% borax, 10.0 wt% HLC, 5.0 wt% DPH, 0.2–0.5 wt% C_3_MimNBr; PBDSN-1 (Gel-1): 0.2 wt% C_3_MimNBr, PBDSN-2 (Gel-2): 0.3 wt% C_3_MimNBr, PBDSN-3 (Gel-3): 0.5 wt% C_3_MimNBr). With the same preparation steps, the hydrogels of PB (10.0 wt% PVA and 0.5 wt% borax), PBD (10.0 wt% PVA, 0.5 wt% borax and 10 wt% HLC), PBDS (10.0 wt% PVA, 0.5 wt% borax, 10 wt% HLC and 5.0 wt% DPH) were obtained. The solutions of BDN (0.5 wt% borax, 10.0 wt% HLC, 0.3 wt% C_3_MimNBr) and DSN (10.0 wt% HLC, 5.0 wt% DPH, 0.3 wt% C_3_MimNBr) were obtained.

Freeze drying of hydrogels: The hydrogel was pre-frozen at −20 °C in a refrigerator for 12 h, followed by vacuum freeze drying in a lyophilizer (LD-DG10, Shandong Holder Electronic Technology Co., Ltd. Weifang, China) for 48 h to completely sublimate internal water. The freeze-dried hydrogel xerogel was collected for further structural and morphological characterizations.

### 4.4. Characterization

^1^H NMR Measurements: ^1^H NMR spectra of samples were obtained by a Bruker ADVANCE 400 spectrometer (Billerica, MA, USA) equipped with a pulse field gradient module (*Z*-axis) using a 5 mm BBO probe. The instrument was operated at a frequency of 400.13 MHz at 25 ± 0.1 °C. All samples were dissolved in DMSO-*d*_6_.

Tube Inversion Method: The tube inversion test was used to qualitatively characterize the sol–gel transition behavior of the hydrogel precursors. Briefly, the prepared precursor solution was loaded into sealed glass vials and incubated under preset conditions for a certain period. Subsequently, the vials were inverted vertically, and the flow state of the internal sample was recorded by digital photography. The sample that remained stationary without downward flow was defined as a successfully formed stable hydrogel network.

Fourier Transform Infrared (FTIR) Spectroscopy: The FTIR spectra of freeze-dried hydrogels were measured on a Thermo Fisher Infrared Spectrometer (Thermo Nicolet iS5, Shanghai, China). The experiments were performed by using the blocks/films preparing technique over a range of 4000–400 cm^−1^ with a resolution of 4 cm^−1^ and total of 32 scans for each sample.

Ultraviolet–Visible (UV-Vis) Spectroscopy: The UV-Vis absorption spectra were recorded on a UV-Vis spectrophotometer (Shimadzu UV-2700, Tokyo, Japan). All samples were diluted with deionized water to a uniform concentration before testing. The scanning wavelength was set from 200 nm to 400 nm with a constant scanning rate, and pure deionized water was used for baseline correction. Quartz cuvettes were adopted for liquid testing, and the absorbance value at each wavelength was automatically collected to obtain the final absorption curves.

X-ray Diffraction (XRD) Measurement: The structure of the freeze-dried hydrogels was measured on a DMAX-2500PC diffractometer (Rigaku Corporation, Tokyo, Japan) with a Cu Kα radiation source (λ = 0.15418 nm).

Rheological Measurements: The rheological properties of the hydrogels were performed at 25.0 °C on an Anton-Paar Rheometer (Graz, Austria). The experiments were measured by a core-plate system of C35/1° Ti L07116 with a plate diameter of 35 mm and core angle of 1°. In the steady-state shear tests, the shear rate over a range rates from 0.01 s^−1^ to 1000 s^−1^ at a frequency 1 Hz. In frequency sweeping tests, the frequency sweeping was from 0.01 Hz to 100 Hz at a shear rate of 1 Pa. In addition, the crossover frequency, f_cross_, is defined as the frequency at which the storage modulus (G′) equals the loss modulus (G″), corresponding to the sol–gel transition point. The relaxation time (τ_relax_), which corresponds to the average crosslink lifetime, can be derived from τ_relax_ = 2π/ω_c_ = 1/f_cross_.

Self-Healing Experiments: The self-healing property was measured by rheological tests. The obtained storage modulus (G′) and loss modulus (G″) were assessed as the self-healing capacity of the samples [36]. For self-healing property tests, fixing frequency at 1 Hz, time sweeps were employed to evaluate the self-healing capability of the hydrogels. The self-healing property of the hydrogel was evaluated via recovery strain tests, which was performed as follows: γ (strain) = 10% (150 s) ⟶ γ = 200% (150 s) ⟶ γ = 10% (150 s) ⟶ γ = 200% (150 s) ⟶ γ = 10% (150 s) ⟶ γ = 200% (150 s) at frequency 1 Hz.

Additionally, the self-healing ability of the hydrogel was evaluated by a cutting healing experiment. The PBDSN (Gel-2) hydrogel was cut in half, after which the halves are crisscrossed with each other and touched together. Upon standing at room temperature for 5 min, two pieces of the broken hydrogel healed into a complete hydrogel without any external stimulation (Figure 4b). The self-healing behaviors of PBDSN (Gel-2) hydrogel were further investigated by non-in situ healing tests. Two hydrogels were separately stained with methylene blue and eosin. During the self-healing process, the unstained hydrogel and the two stained hydrogels were brought into contact in air, without applying any external stress or stimulus [37]. The interface between the two stained hydrogels was then observed under a microscope at different time intervals to assess their self-repairing performance. The morphological changes in the hydrogels under all conditions were recorded by photography.

Field Emission Scanning Electron Microscopy (FE-SEM): To investigate the morphologies and microstructural characteristics of hydrogels, Field Emission Scanning Electron Microscopy (JEOL JSM-6700F FE-SEM, Tokyo, Japan) was employed. Hydrogel sample was first freeze-dried under vacuum for 24 h using a freeze dryer. A small volume of xerogel was affixed on silica wafers, after which the samples were coated with a layer of gold and observation using the SEM.

In Vitro Antibacterial Activity Evaluation: In vitro antibacterial activities of the hydrogels against Gram-positive bacteria *Staphylococcus aureus* (*S. aureus*, ATCC 25923) and Gram-negative bacteria *Escherichia coli* (*E. coli*, ATCC 25922) were employed as the model bacterium for in vitro antibacterial assays via plate counting method [44]. The glycerol preserved stock stored at −80 °C was thawed at room temperature. A 100 μL aliquot of the bacterial suspension was streaked onto nutrient agar plates. Following the appearance of single colonies, a single colony was inoculated into Luria–Bertani (LB) and incubated overnight at 37 °C with agitation. The culture was repeatedly streaked for three consecutive passages to ensure full activation. The final log-phase culture was maintained at 4 °C. The activated bacterial cells were harvested by centrifugation at 4000 rpm for 10 min, and the supernatant was removed. The cell pellet was resuspended in sterile PBS and adjusted to a density of 10^8^ CFU/mL. The bacterial working solution was stored at 4 °C until further use.

The formed hydrogels were sterilized by ultraviolet in a biosafety cabinet for 30 min in advance. After, hydrogel (1 g) was put into bacteria culture tube with liquid nutrient medium and ~10^6^ CFU/mL (confirmed by OD_600_ measurement) of bacteria, which was shocked at 37 °C for 12 h in shaker. Then, 100 μL liquid nutrient medium was transferred onto the surface of the Luria–Bertani (LB) plate. The agar plates were incubated at 37 °C for 12 h in an incubator. The colony formation units of agar plates were measured by plate counting method, which reflected the antibacterial activity of hydrogels.

The colony forming units (CFUsample) and (CFUcontrol) of bacteria are quantified in the presence or absence of hydrogel. The antibacterial ratio was calculated using the following formula [45]:

(1) $$\mathrm{Bacteriostasis}\;\mathrm{rate}\;(\%)\;=\;\frac{CFUcontrol-CFUsample}{CFUcontrol}\times 100\%$$

## Data Availability

The original contributions presented in this study are included in the article. Further inquiries can be directed to the corresponding author.
